# Data sharing for novel coronavirus (COVID-19)

**DOI:** 10.2471/BLT.20.251561

**Published:** 2020-03-01

**Authors:** Vasee Moorthy, Ana Maria Henao Restrepo, Marie-Pierre Preziosi, Soumya Swaminathan

**Affiliations:** a Science Division, World Health Organization, avenue Appia 20, 1211 Geneva 27, Switzerland.; b Health Emergencies Preparedness and Response, World Health Organization, Geneva, Switzerland.; c Universal Health Coverage/Life Course, World Health Organization, Geneva, Switzerland.

Rapid data sharing is the basis for public health action. The report from the 30 January 2020 International Health Regulations (2005) Emergency Committee regarding the outbreak of novel coronavirus (COVID-19) stressed the importance of the continued sharing of full data with the World Health Organization (WHO). The information disseminated through peer-reviewed journals and accompanying online data sets is vital for decision-makers.^1^^–^^3^ For example, the release of full viral genome sequences through a public access platform and the polymerase chain reaction assay protocols that were developed as a result made it possible to accurately diagnose infections early in the current emergency.

Deficiencies in data-sharing mechanisms – highlighted during the 2013–2016 Ebola virus disease outbreak in west Africa – brought the question of data access to the forefront of the global health agenda.^2^ In September 2015, agreement was reached on the need for open sharing of data and results, especially in public health emergencies.^2^ Subsequently, the International Committee of Medical Journal Editors confirmed that pre-publication dissemination of information critical to public health will not prejudice journal publication in the context of health emergencies declared by WHO.^4^ Furthermore the committee stated that information critical for public health is to be shared with WHO before publication^5^ – a commitment echoed by several leading journals in the context of the COVID-19 response.

Efforts for expedited data and results reporting should not be limited to clinical trials, but should include observational studies, operational research, routine surveillance and information on the virus and its genetic sequences, as well as the monitoring of disease control programmes.

To improve timely access to data in the context of the COVID-19 emergency the *Bulletin of the World Health Organization* will implement an “COVID-19 Open” data sharing and reporting protocol, which will apply during the current COVID-19 emergency.

On submission to the *Bulletin*, all research manuscripts relevant to the coronavirus emergency will be assigned a digital object identifier and posted online in the “COVID-19 Open” collection within 24 hours while undergoing peer review. The data in these papers will thus be attributed to the authors while being freely available for unrestricted use, distribution and reproduction in any medium, provided that the original work is properly cited as indicated by the Creative Commons Attribution 3.0 Intergovernmental Organizations license (CC BY IGO 3.0). Should a paper be accepted by the *Bulletin* following peer review, this open access review period will be reported in the final publication. If a paper does not meet the journal’s requirements after peer review, authors will be free to seek publication elsewhere. If the authors of any paper posted with the *Bulletin* in this context are unable to obtain acceptance with a suitable journal, WHO undertakes to publish these papers in its institutional repository as citable working papers, independently of the *Bulletin*. The choice of a pre-print platform remains the sole discretion of the author. This early access to research manuscripts at WHO builds on examples of other rapid information access platforms such as PROMED and F1000Research.^5^^,^^6^

Given the many unanswered questions on the reservoir, transmission, consequences and manifestations of COVID-19 infection and associated disease, our goal is to encourage all researchers to share their data as quickly and widely as possible. With this protocol for immediate online posting, we are providing another means to achieve immediate global access to relevant data. By submitting their studies to “COVID-19 Open,” researchers can share their data while meeting their need to retain authorship, document precedence and facilitate international scientific cooperation in the response to this emergency.
